# Tricuspid Valve Endocarditis Beyond the Usual Suspects in a Hemodialysis Patient: A Case Report

**DOI:** 10.7759/cureus.101169

**Published:** 2026-01-09

**Authors:** Saketh Parsi, Kunal Sonavane, Harikrishna Choudary Ponnam, Laxmi Sakamuri, Pallavi Shirsat

**Affiliations:** 1 Hospital Medicine, Ascension Seton Medical Center Austin, Austin, USA; 2 Internal Medicine, Willis Knighton Health, Bossier City, USA; 3 Internal Medicine, Summa Health, Akron, USA; 4 Nephrology, Minden Medical Center, Minden, USA

**Keywords:** arteriovenous (av) fistula, infective endocarditis, maintenance hemodialysis, mssa bacteremia, tricuspid valve vegetation

## Abstract

Right-sided infective endocarditis (IE) is predominantly less prevalent than left-sided IE in hemodialysis (HD) patients because the left side is attributed to valvular damage from high-pressure circulation, endothelial vascular differences, and exposure to oxygenated blood. Right-sided IE is most commonly associated with intravenous drug use, central venous catheters, or intracardiac devices. However, it is associated with an increased mortality in HD patients. We present a case of an HD patient with no arteriovenous fistula (AVF) infection or history of intravenous drug use, who presented with right-sided IE. A 33-year-old man with a history of diabetes mellitus and end-stage renal disease on HD via an AVF presented with fever and chills after undergoing thrombectomy for right upper extremity brachial vein deep vein thrombosis, so he was discharged on two weeks of vancomycin administered with dialysis. The patient returned two weeks later for recurrent fever despite adherence to his antibiotic therapy. Blood cultures from previous and current admission grew methicillin-sensitive *Staphylococcus aureus* (MSSA), prompting echocardiography, which demonstrated tricuspid valve (TV) vegetation. The patient was transferred to a tertiary center and underwent endovascular mechanical aspiration of the TV vegetation by the cardiothoracic surgery team. His symptoms were resolved, and he was discharged on a six-week course of intravenous vancomycin 1 gram with hemodialysis and outpatient follow-up. Our case highlights a rare instance of TV vegetation in an HD patient with rapid onset of MSSA bacteremia following thrombectomy, despite the absence of AVF infection or other risk factors, suggesting hematogenous seeding during the procedure or AVF cannulation. We conclude that right-sided IE, particularly TV vegetation, should be considered in HD patients with persistent bacteremia, even in the absence of AVF infection or other identifiable causes of right-sided IE.

## Introduction

Right-sided infective endocarditis (IE) is uncommon, accounting for only 5-10% of all IE cases, with tricuspid valve involvement representing approximately 90% of these episodes [1]. IE carries a significantly higher mortality rate among hemodialysis (HD) patients compared to the general population, with the risk particularly elevated in those using HD catheters rather than arteriovenous fistulas (AVFs) [2].

Although HD patients are frequently exposed to episodes of bacteremia due to repeated percutaneous needle punctures and vascular access through AVFs, right-sided IE remains relatively rare in this population. Higher rates are observed instead among individuals who inject drugs intravenously (IV), those with long-term central venous catheters, or patients with implanted cardiac devices [1,2]. The low incidence of right-sided IE in HD patients may be attributed to several factors. High-velocity blood flow through a well-functioning AVF may limit bacterial adhesion to right-sided valves. Additionally, the predominantly staphylococcal pathogens encountered in this population are less likely to involve right-sided valves compared with settings such as IV drug use [3]. Left-sided IE is more common among HD patients, likely due to differences in endothelial vascularity, the low oxygen content on the right side of the heart, and valvular damage associated with the high-pressure systemic circulation. This damage increases susceptibility to valvular calcification secondary to disturbances in calcium-phosphorus homeostasis, as well as cardiovascular stress and injury during HD [4,5]. The most common causative organism in HD-related IE is *Staphylococcus aureus*, followed by *Enterococcus *species [6]. We report a rare case of right-sided infective endocarditis in a hemodialysis patient with no central venous catheter or evidence of arteriovenous fistula infection or history of IV drug use.

## Case presentation

A 33-year-old African American male with a history of hypertension, type 1 diabetes mellitus, hypothyroidism, and legal blindness secondary to diabetic retinopathy, end-stage renal disease (ESRD) on scheduled HD (Monday, Wednesday, Friday) via a left upper-extremity AVF, was referred from the dialysis unit for evaluation of subjective fevers since one to two days before this admission, associated with upper respiratory symptoms, and nasal congestion, which resulted in a missed HD session. Three days prior, he had undergone an unsuccessful thrombectomy for a right upper extremity brachial vein deep venous thrombosis (DVT) and had been discharged on anticoagulation therapy. 

On physical examination (PE), blood pressure (BP) was 100/57 mmHg, temperature (T) 98.5°F, heart rate (HR) 99 beats/min, and respiratory rate (RR) 20 breaths/min. His AVF demonstrated a palpable thrill without overlying erythema or tenderness. Initial laboratory evaluation revealed leukocytosis with a white blood cell (WBC) count of 13,200 cells/mm³ and a hemoglobin level of 7.3 g/dL (Table 1). Nephrology was consulted, and the patient underwent hemodialysis with concurrent vancomycin therapy. He refused to stay overnight, so he was discharged on IV vancomycin 1 gram administration during HD sessions for two weeks.

**Table 1:** Laboratory values during the patient’s hospital stay. CRP, C-reactive protein; Cl, chloride

Lab test	Normal value	First admission	Second admission		
		Day 1	Day 1	Day 2	Day 3
White blood cells	4000 to 11000 cells/mcL	13200	19500	20300	17600
Hemoglobin	13.5 to 17.5 g/dL	7.3	8.1	8.4	8.4
Platelets	150 to 450 K cells/mcL	230	361	339	302
CRP	<0.3 mg/dL	-	16.8	-	-
Sodium	135 to 145 mEq/L	133	130	128	135
Potassium	3.5 to 5 mEq/L	4.3	4.6	4.9	3.6
Cl	96 tp 106 mEq/L	94	88	86	95
Urea nitrogen	8 to 24 mg/dL	46	49	54	30
Creatinine	0.7 to 1.3 mg/dL	10.6	11	11.5	7.3
Procalcitonin	<0.05 ng/mL	50.3	97.6	-	-

He returned two weeks later with persistent fevers with a Tmax of 104 °F. On PE, BP: 109/57 mmHg, HR: 79 beats/min, RR: 18 breaths/min, T: 99.8°F. Blood cultures from his prior admission were noted to be positive for methicillin-sensitive *Staphylococcus aureus *(MSSA). Repeat laboratory evaluation revealed worsening leukocytosis (WBC: 20,300 cells/mm³), hemoglobin 8.4 g/dL, C-reactive protein 16.8 mg/dL, and severe hyperglycemia with a glucose level of 519 mg/dL. His hyperglycemia was managed with insulin lispro sliding scale subcutaneously. He was initially started on IV linezolid 600 mg every 12 hours, which was later transitioned to IV daptomycin 400 mg every 48 hours, in addition to IV piperacillin-tazobactam therapy 4.5 grams every 12 hours. Blood cultures during the second admission also showed the same organism (MSSA).

**Figure 1:**
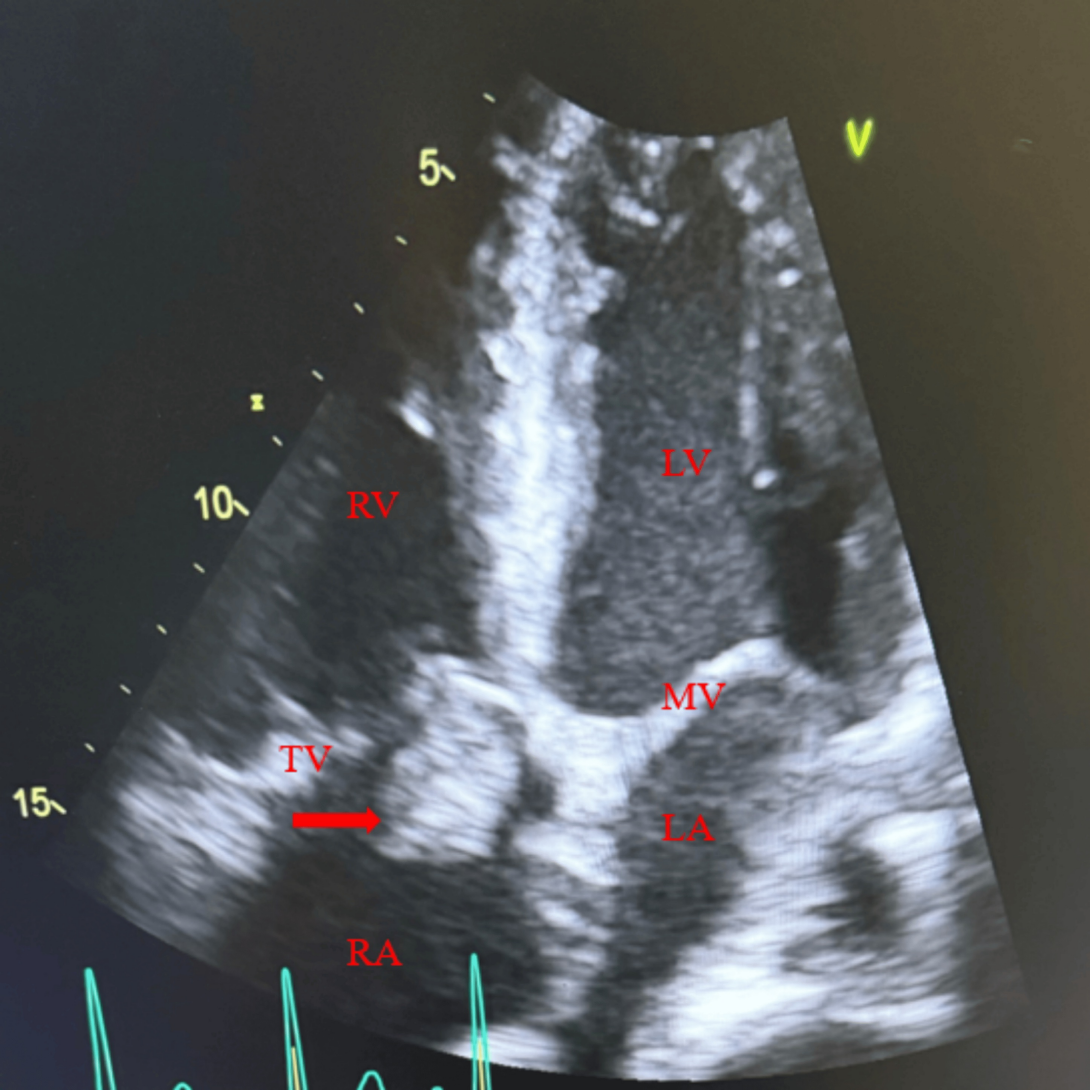
Transthoracic echocardiography, apical four chamber view showing tricuspid vegetation. Red arrow pointing to the tricuspid valve vegetation. RV, right ventricular; LV, left ventricular; TV, tricuspid valve; MV, mitral valve; RA, right atrium; LA, left atrium.

A chronic non-healing wound located posterior to the left ear, with concern for possible mastoid osteomyelitis, was initially suspected as the source of MSSA bacteremia. However, due to persistent bacteremia despite antimicrobial therapy, a transthoracic echocardiogram was obtained and demonstrated a large mobile tricuspid valve (TV) vegetation, associated with moderate eccentric jet of tricuspid regurgitation (TR) (Figure 1), confirming the diagnosis of right-sided infective endocarditis. Cardiology was consulted, and after multidisciplinary discussion with the cardiothoracic surgery team, the patient was transferred to a tertiary center where he underwent catheter-directed removal of the TV vegetation. He was subsequently discharged with a plan to complete six weeks of IV vancomycin 1 gram administered with his HD. He completed the course of antibiotics and remained fever-free when followed up six months later.

## Discussion

We present a case of a rare but high-risk condition: right-sided IE, specifically TV vegetation, in an HD patient with no history of intravenous drug use, cardiac device implantation, or arteriovenous fistula infection, who underwent catheter-directed vegetation removal.

The prevalence of IE among HD patients is approximately 2.9%, and the incidence is 50-60 times higher in this population compared with the general population. Among HD patients who develop IE, left-sided involvement predominates, accounting for 80-100% of cases [4]. This predominance is attributed to the high-pressure systemic circulation, which predisposes to valvular damage, progressive calcification, and disruption of endothelial integrity [4]. AVF-related infections occur in 2-4% of HD patients, with an incidence of 0.018 per 100 access days; however, although AVF infections can occasionally precipitate IE, this mechanism was not present in our patient [7]. In contrast, right-sided IE is relatively uncommon in HD patients and is typically associated with intravenous drug use, indwelling cardiac devices, or HD catheters [8]. Notably, our patient had no history of intravenous drug use or cardiac device in situ or catheter dependence, making the presentation atypical.

*Staphylococcus *and *Streptococcus *species remain the most frequently isolated pathogens in IE, followed by *Enterococcus *species. *Staphylococcus aureus *is the most common causative organism in HD-related IE [9]. This was consistent with our patient, whose blood cultures grew *S. aureus*. IE in HD patients is associated with significantly higher morbidity and mortality compared with the general population [10], approximately 30% of HD patients develop sepsis, with an incidence of 12.66 episodes per 100 person-years, occurring most commonly in catheter-dependent patients and least frequently in those with AVF [11]. Therefore, early recognition and timely initiation of appropriate therapy, together with a coordinated multidisciplinary approach, are essential, as demonstrated in our case.

Bacteremia associated with intravascular catheters or cardiac devices is typically managed by removing the infected hardware [12]; however, this mechanism was not applicable in our patient, who had neither an indwelling catheter nor a cardiac device. Most cases of right-sided IE respond favorably to conservative medical management; nevertheless, approximately 20-25% of patients ultimately require surgical intervention [13]. Established indications for surgical management include: (a) severe right-sided heart failure secondary to significant tricuspid regurgitation; (b) persistent bacteremia despite appropriate antibiotic therapy for more than seven days; (c) large tricuspid valve vegetations measuring >20 mm; and (d) recurrent septic pulmonary emboli [14].

In surgically appropriate candidates, management of tricuspid valve endocarditis typically includes tricuspid valve repair, replacement, or valvectomy. However, catheter-directed aspiration of right-sided vegetations is preferred in patients who are at high risk for open-heart surgery [15]. For this reason, catheter-directed TV vegetation removal was pursued in our patient.

## Conclusions

Clinicians should maintain a high index of suspicion for bacteremia and right-sided infective endocarditis in hemodialysis patients presenting with signs of sepsis, even in the absence of intravenous drug use or AVF infection. To improve prognosis and reduce adverse outcomes, early recognition is crucial, as right-sided endocarditis may arise from non-traditional sources in this population, underscoring the importance of targeted antimicrobial strategies, uniform infection control protocols, and careful consideration of patient-specific risk factors. Additionally, a multidisciplinary approach integrating nephrology, cardiology, and cardiothoracic surgery is critical in the management of hemodialysis patients with suspected or confirmed tricuspid valve endocarditis, given its high morbidity and mortality, and further research should concentrate on ESRD-adapted sepsis diagnostic and interventional models to reduce infection-related mortality in this high-risk population.
